# Food Design Thinking: A Systematic Review from an Evolutionary Perspective

**DOI:** 10.3390/foods13152446

**Published:** 2024-08-02

**Authors:** Ana Castanho, Carla Brites, Jorge C. Oliveira, Luís M. Cunha

**Affiliations:** 1 GreenUPorto/INOV4Agro, DGAOT, Faculty of Sciences, University of Porto, 4485-646 Vila do Conde, Portugal; anavargascastanho@gmail.com; 2 Instituto Nacional de Investigação Agrária e Veterinária, I.P. Av. da República, Quinta do Marquês, 2780-157 Oeiras, Portugal; 3 GREEN-IT Bioresources for Sustainability, ITQB NOVA, Av. da República, 2780-157 Oeiras, Portugal; 4 School of Engineering and Architecture, University College Cork, College Road, T12 YN60 Cork, Ireland

**Keywords:** Food Design Thinking, design theory, design thinking application, PRISMA

## Abstract

Design thinking (DT) has been a subject of extensive debate and application across diverse knowledge domains, including the realm of food; nonetheless, its precise definition remains unclear. This systematic review comprised two components. Firstly, it examined the evolving understanding of DT by aggregating pertinent studies selected based on their representativeness, determined by the volume of citations. This process was deployed using citation mapping software, complemented by an analysis of the most pertinent reviews within this domain. Secondly, it investigated the Food Design Thinking (FDT) approach. The review encompassed a total of 22 references and reviews in the first segment and 27 studies in the second segment. In Part 1, the results revealed the emergence of two principal areas of investigation, namely education and management, stemming from the foundational DT theory. Furthermore, the findings highlighted that DT has assimilated the knowledge gathered from these domains. In Part 2, the outcomes illustrated the utilisation of FDT to address a multitude of food-related issues, including education, sustainability, health and wellbeing, and the development of food products. From this analysis, it is notable that this approach presents contextual variations while emphasising the notion of integration of the consumers throughout the FDT process.

## 1. Introduction

The expression “design thinking” (DT) has been increasingly referred to in the literature. In searches for the expression in the most relevant academic databases, such as Scopus or Web of Science, between 2005 and 2024, it is possible to verify that the published works show a positive growth rate. Furthermore, in analyses of the search results, it is possible to confirm that the expression is commonly used in various research areas, such as engineering, computer science, education, economics, social sciences, or healthcare. Although in food studies and the associated fields of knowledge (e.g., nutrition or food engineering), the expression is not so present as in the knowledge areas mentioned above, some authors [1,2] have noted that DT could also be useful for food innovation.

As already exposed by others [3,4,5], the expression DT is used in divergent ways as a transdisciplinary and user-centred method directed to innovation and popularised by IDEO and Stanford d.School [6,7], or as a part of the designers’ activity discussed by design theorists and authors such as [8,9,10,11].

While this discussion regarding DT is relatively recent, the concept of design, particularly the ways of thinking about design and design problems, was also discussed previously [12]. For Alexander [13], design is about doing physical things (giving form, organisation and order, working in the concrete realm); for Simon [11] it is the “knowledge” that allows human action to respond to problems, working in the abstract realm. Traditionally, the designer was seen as an artefact maker, and the design and the making co-occurred; as the concept evolved, the making of artefacts and the DT separated into different realities, where the making is usually completed after the thinking process [14].

While some divergence may contribute to a healthy discussion and the concept’s evolution, the DT concept, particularly the simplistic way it is used and what it promises, is seen by others as a fad, as McCausland [15] reported. In her speech at the Adobe 991 Conference, the designer Jen [16] criticised DT, pointed out the simplistic way the practice of design is summarised in a five-step linear diagram, and how the design theory authors are excluded from the current DT approach [16]. In his blog, the designer Ricardo Martins expressed his ideas on why DT is a fad, based on a work by Miller et al. [17], who defined a fad as a series of statements that propose a quick fix for a problem and have a typical life cycle: they become popular quickly, stay popular for a few years, and have an abrupt decline in interest. For Miller et al. [17], there are also common characteristics of management fads, which Martins [18] correlated with DT: a fad is simple, promises too much, and can be applied to any context; it can also be implemented incompletely, which allows managers to believe they are being modern without a final evaluation of the DT strategy’s success. The author stated that DT only reformulates old ideas more appealingly, in accordance to the zeitgeist, and although it was created by theorists, a well-known company popularised it. Bruce Nussbaum, one of the advocates of DT, considers “the success rate for Design Thinking processes was very low”, and the expectations about how DT could be a linear methodology that could deliver innovation did not meet reality [19]. Michael Hendrix, an IDEO partner, stated that many people use DT in “superficial ways”, turning it into a “theatre of innovation”, and argued that DT is not a formula but is instead a set of “milestones in the creative process”, and its success depends on the company’s culture [20].

The use of DT in food studies is relatively new; however, some authors have advocated that the use of the Food Design Thinking (FDT) approach can benefit innovation in the field by integrating the consumer not only as a validator but as a point of departure for innovation; furthermore, FDT also presents other advantages in this domain, as it is claimed to promote faster results through rapid prototyping and a more hands-on approach [2,21].

Citing Liedtka [22], “bringing rigorous empirical scrutiny to the design-thinking process could itself be construed as a wicked problem: the problem can be defined in many different ways”. Considering the difficulties inherent to the definition of the DT concept, this review aimed to identify the roots of DT from a historical perspective based on its academic citation network to better understand the importance of this approach to the realm of food.

## 2. Methodology

The research methodology was divided into two parts: (1) a review of the roots of DT and (2) a review of the FDT approach. The research mentioned in this article was carried out during the first quarter of 2024.

**Figure 1:**
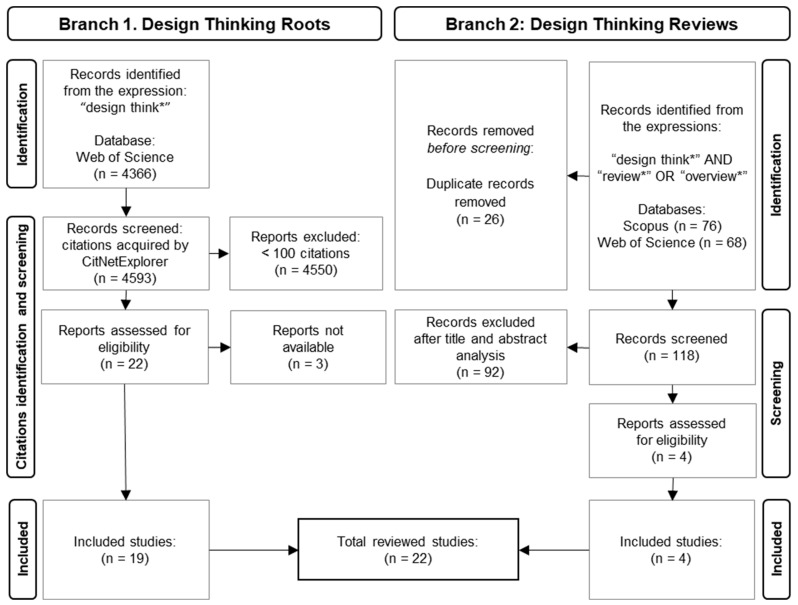
Schematic view of the methodology used to identify and screen the most cited works regarding Design Thinking (Branch 1), and the PRISMA methodology used to obtain the most relevant reviews.

In the first part (Figure 1), the research was based on the most cited works and was divided into two research branches. The first branch aimed to identify the most cited works in the DT literature. To determine the most cited works, research based on the expression “design think*” was conducted using the Web of Science (WOS) search engine, considering its presence in the title, abstract, or keyword fields. The references were extracted as “full references”, which included citations; thus, the retrieved database consisted of the original text and the cited works. Both original and works cited in the references presented some inconsistencies in some fields; for instance, some authors’ names had different spellings (e.g., Simon A.H., Simon H., or Simon H.A.); therefore, entries for those fields were standardised by using Visual Studio Code v. 1.8 as database code editor to find, replace, or correct the inconsistent data. The standardised data were explored using the citation network manager CitNetExplorer v. 1.0.0 (Leiden University). The most cited references (>100 citations) were used as data sources for the review.

The second branch of the first part consisted of selecting relevant reviews to complement and validate the data found in the first branch. The research and selection for review articles regarding DT was performed by using the Prisma 2020 Statement method [23] by retrieving the works from the WOS and Scopus databases, starting from the expression “design think*” AND “review*” OR “overview*” (in the title, keywords, and abstract). In total, 118 review articles were screened after carefully reading the title and abstract. Several works (*n* = 92) were removed in the screening phase due to several factors, such as being out of scope, as the expression only appeared as keywords; in other cases, DT was only used superficially, not being the central theme of the review. After joining the two branches, 23 studies were selected for review in Part 1.

**Figure 2:**
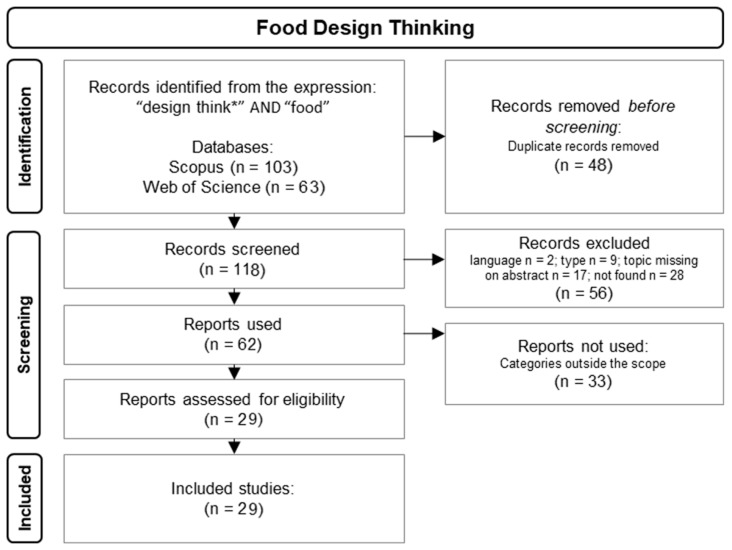
Schematic view of the PRISMA methodology used to obtain the data for Part 2.

For Part 2, a search for “design think*” AND “food” (including the title, keywords, and abstract) was performed in WOS and Scopus during the first quarter of 2024. The Prisma 2020 Statement method [23] was used to screen the references. The refinement of the obtained works is shown in Figure 2. From the 116 original articles, 56 were excluded in the first phase, as the abstract did not contain the expression “Design Thinking” (*n* = 17), the articles were not in English (*n* = 2), they were not research articles (*n* = 9), or they were not accessible (*n* = 28). From the remaining documents (*n* = 60), 33 were removed after a second screening: the abstract was carefully read, and 33 articles were excluded due to FDT not being the article’s central theme. The 27 remaining articles were grouped according to the subject and content analysed.

## 3. Results and Discussion

### 3.1. Methodological Notes

Due to the incredible popularity of DT and its inclusion in many areas as an “umbrella concept” [4], works on the theme abound. Considering this particularity, the approach to the first phase intended to capture the essence and the evolutive course of *what DT is*. Due to the intense discussion on the subject, with some designers arguing against the “management DT approach”, labelling it as a “*fad*” [15], the applied identification of studies and screening methodology for the branch of the first part intended to minimise the researcher bias by including the most referred works used in the theme with little human intervention, being guided by a citation network manager (CitNetExplorer version 1.0.0). Although CitNetExplorer only worked with references from WOS, due to technological limitations, the advantages of it being a simple and objective tool were considered more important in this scenario; furthermore, this approach allowed us to explore relevant works that, despite not being indexed to scientific databases, consisted of the most essential works on the theme (considering academic works on design; moreover, the older ones are based on books with no experimental data). Since the method could present limitations, such as missing essential pieces or concepts, a collection of reviews was analysed to complement the work.

The second part of the study revealed how the expression “DT” was used by many authors to promote their studies, taking advantage of the DT trend. This can be observed by the number of studies collected that passed the first screening phase and were discarded. Many of the studies used “design thinking” as a keyword or in the title, even though the theme would not be the focus of the work; in some cases, the exact wording did not appear in the whole article. This might be one of the causes of DT being considered a fad.

### 3.2. Part 1. The Roots of Design Thinking 

The references included in the citation analysis and the selected reviews are presented in Table 1, grouped by chronological order and theme, representing the order of this review.

#### 3.2.1. Design Thinking

The Sciences of the Artificial, by Simon [11], has significantly impacted the literature across various knowledge fields and is considered “one of the most influential texts in the 50-year history of the development of design theory” [39]. Since its first publication, it has been revisited by numerous authors [39,40,41], and updated and republished in 1981 and 1996. To better understand the impact of The Sciences of the Artificial, it is essential to note that its first publication coincided with the beginning of the third industrial revolution, a period marked by a fascination with computers, automation, and a desire to “scientise”. When applied to design, this *zeitgeist* led to “the design methods movement” in the 1960s [39].

Simon [11] described design through the concept of the artificial, distinguishing it from natural phenomena. This distinction underscores the difference between science and engineering: while science observes and analyses *what things are*, engineering is concerned with *creating the artificial* or achieving a specific goal and function. For Simon [11], the natural and the artificial interact as a system: artificial things can be made of natural materials, and the artificial influences the natural world and vice versa. This interaction is mediated by the artefact, which serves as the interface between the system’s interior and exterior. Thus, the designer’s role is to draw this boundary, which functions as a homeostatic mechanism.

Simon’s theorisation led to the concept of design as “the science of the artificial”, similar to the “natural sciences”. The designer is thus seen as someone who transforms “existing situations into preferred ones”, implying a problem-solving approach that broadens the definition of a designer, bringing professions such as engineering, architecture, business, education, law, and medicine into the field of design. While Simon [11] proposed a “*scientifisation*” of design, the author believed a set of problems could not be solved linearly. Regardless of sharing Simon’s view [11] on the distinction between natural and artificial, Rittel and Webber [24] focused on the nature of design problems. In the article “Dilemmas in a general theory of planning”, Rittel and Webber [24] defined design problems as “wicked” and argued that they cannot be solved in the linear way science proposes. For Rittel and Webber [24], natural sciences’ problems are well-defined, separable, and have findable solutions. In contrast, the problems of design need to find the problem’s definition (the difference between the observed and the desired) and the problem’s location (where the problem is), thus being “wicked”.

Schön [25] continued defining design by comparing it with natural sciences in his book The Reflective Practitioner: How Professionals Think in Action. However, the author focused on how designers think while criticising how technical rationality sees design. While earlier authors (e.g., Simon [11]) focused on the solution, the decision-making process, the objectives to meet, and the means to reach them, Schön [25] introduced the design process as a process of reflection in action. Reflection in action requires a constant reframing of the problem and a continuous understanding of the interactions between the means and the end, needing to adapt to the situation as unexpected results emerge, as evident in professions such as clinical psychology, design, and architecture.

While for Schön [25], the design process is based on a dialogue between the designer and the situation, Rowe [26] approached it as a more structured process, such as the scientific method [42]. On the basis of his experience with architecture students, Rowe [26] described DT as a decision-making process, denying the idea of an ideal step-by-step sequence. Instead, Rowe [26] suggested that design follows an episodic structure, described as a “movement back and forth between areas of concern”, with periods of speculation and contemplation. Rowe [26] tried to identify a generic problem-solving procedure and to understand the source of the concepts that construct the frameworks. For Rowe [26], the answer lies not only in the problem-solving methods but in the frames applied to them. This constant reframing of problem-solving leads to uncertainty, which is both frustrating and joyful for the designer. Although these rapid changes are essential to the design process, as the conflicts often stimulate creativity [30], they require the designer to have some specific traits, such as being aware and sensitive to environmental nuances, noticing coincidences, recognising opportunities, identifying favourable conjectures, becoming profoundly involved, or being optimistic. Despite the importance of personality and personal motivations, designers can exercise their skills [26], which have been studied by other authors.

**Figure 3:**
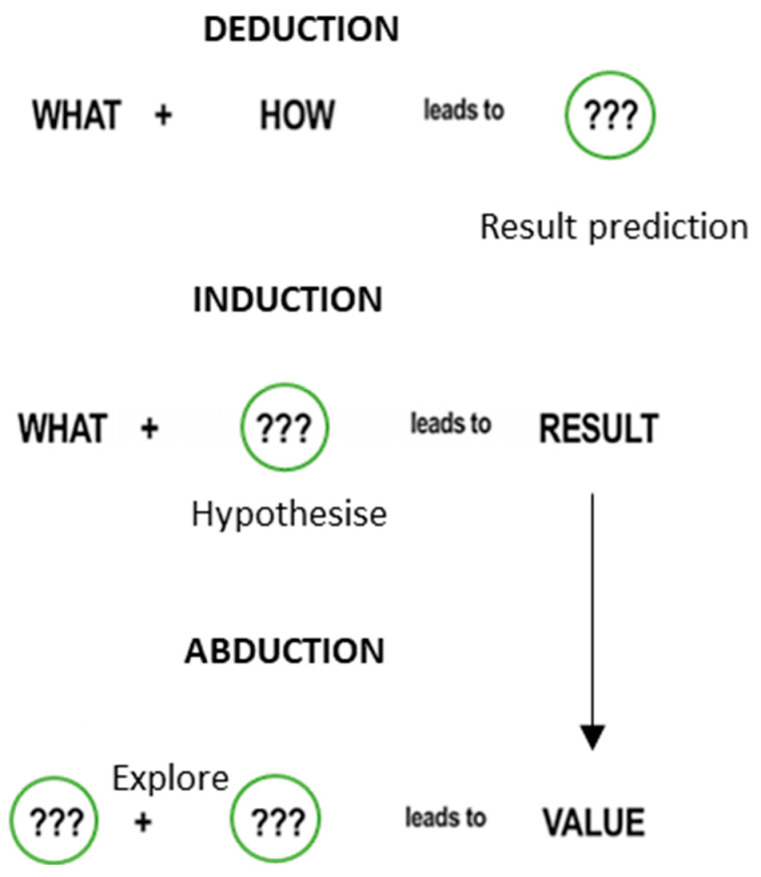
Inductive, deductive, and abductive reasoning based on Aristotelian logic. Adapted from Dorst [27].

Dorst [27] described the DT framework using concepts of formal logic based on Aristotelian logic such as induction, deduction, and abduction. Deductive reasoning is a “way of thinking that relates ideas to one another in reaching conclusions”. In contrast, inductive reasoning “uses a specific observation to reach a general conclusion” [43]. According to Peirce’s theory [44], abduction is “the process of forming explanatory hypotheses”. It includes “all the operations by which theories and conceptions are engendered”. To better understand these concepts, Dorst [27] expressed them visually (Figure 3).

Dorst [27] compared the application of framing by novice or expert designers. While students generate proposals for the “how” and the “what” and match them, experts develop “frames”. However, when the problem is a paradox, even an expert needs to keep reframing the problem. Experienced designers start working on a solution only after establishing the core paradox, searching search for clues by broadening the problem’s context. To create new frames, expert designers deal with analysis, creating *themes* or “sense-making tools”. Despite *themes* being outside the core problem, they trigger the creation of new frames, leading to a solution. Thus, personal traits, training in skills, and experience contribute to how designers solve problems [26].

#### 3.2.2. Design Thinking as a Liberal Art

Although Simon [11] already expanded the discourse of design to other areas, Buchanan [8] popularised it, referring to design as “the new liberal art” or a flexible area that could expand and integrate very different fields of knowledge, and also being present in daily lives, leading to a productive end. The idea of the expansion to other areas, as proposed by Buchanan [8], materialised as DT was integrated into design education and management. Dym et al. [29] discussed the importance of design in engineering education, as engineering leads to “processes whose form and function have to achieve clients’ objectives or users’ needs while satisfying a specified set of constraints”. Therefore, DT should be included in the context of engineering by (a) including divergent–convergent thinking (where divergent thinking, a major component of creativity, stands for the production of many solutions to a problem, and convergent thinking is for the synthesis of the alternatives into a solution [45]) to deal with wicked problems, (b) applying the process of framing to decision-making, (c) promoting teamwork and user-centred design; and (d) using both the language of design and engineering by mixing verbal, graphic, and formal representations with numbers or analytic models.

Dorst and Cross [28] experimentally studied the creative process in the context of education, describing design as a matter of developing and refining in an iterative process of analysis, synthesis [39], and evaluation that occurs in two design spaces: the problem and the solution. This idea of co-evolution of the design process was also referred to by other authors [25,26]; the dialogue between the problem and the solution ends when a “problem–solution pair” or “problem framing” is created. The authors introduced the notions of “default” and “surprise”, where the “default” questions are given by the constraints and the “surprise” of the designers, leading to a process of a “burst of development” instead of a gradual change. Cross [30] also explored the differences between novice and expert designers, concluding that novice designers usually identify a problematic aspect and immediately begin exploring its solution in depth. In contrast, expert designers move quickly to early conjectures of the solution and use these conjectures to explore and define the problems and solutions together. The change of focus from the problem to the solution appears to be a feature of design expertise that develops with education and experience in designing [30].

Seidel and Fixson [32] studied the use of brainstorming in multidisciplinary teams, concluding that it resulted in success during the entire process and the integration of new members to the team. On the basis of experimental studies, Razzouk and Shute [31] concluded that including DT in education may better prepare students for real life by teaching them to solve complex problems and develop critical thinking skills.

DT was also used in the management field. The reviewed works on the theme are similar, as the authors shifted the traditional management model to a new one. For Brown [33], the need for this new approach derives from the new societal challenges, where “products” and “services” are being merged, the complexity of problems is increasing, and sustainability is a real issue to be worked on. As a human-centred approach, DT can help deal with that complexity, inspiring solutions with global potential that are part of global activism. The author emphasised the collaborative nature of DT not only as a way of integrating all the participants in the process but also to improve lives in an empathic way, where there is no “us versus them” but “us with them”.

For Martin [34], the core of the dilemma of innovation relies on the tension between reliability and validity, where the goal of reliability is to produce consistent and predictable outcomes, and the goal of validity is to produce outcomes that meet the desired objective; in this dynamic, most companies choose reliability over validity, as it seems safer [35]. DT lies between reliability and validity, between art and science, between intuition and analysis, and between exploration and exploitation by applying abductive reasoning and deductive and inductive logic.

In the new management approach, companies should work as designer shops, working on projects collaboratively and iteratively (as a human-centred approach), using abductive reasoning and seeing constraints as an opportunity. This new approach should be incorporated from the top of organisations and taught in management schools [7,33]. Brown [33] and Martin [34] suggested the use of designers’ tools, such as observation, brainstorming, visual expression (through sketching or sticky notes), storytelling, and prototyping. Prototypes are viewed as essential in the process and have been needed since the beginning to accelerate the exploration of ideas and to set goals. Storytelling is also important, as it inserts the time dimension into the project, working in two ways: it helps to visualise the idea through time, which is helpful to designers, while it can also help communicate the value of an idea to the client at the end of the project.

Martin [34] described the design process as a knowledge funnel with three phases: (1) exploration of the problem, starting with intuition; (2) the heuristic, which transforms the intuition into language; and (3) the algorithm, where the heuristic is transformed in a step-by-step procedure. This process of refinement occurs as a dynamic interplay between innovation and efficiency. For Brown and Wyatt [35], the process starts with a brief, which helps the project team to begin. However, to the authors, it is essential to observe peoples’ experiences. The ideation phase relies on divergent thinking, a process usually conducted by brainstorming in a multidisciplinary and interdisciplinary team; in the generation of ideas, the use of visual means should be encouraged.

#### 3.2.3. Human-Centred Design

Brown [6] describes DT as a human-centred design methodology, where innovation is powered by direct observation of the end user’s needs. The process is described as a system of spaces: inspiration, ideation, and implementation, which are used iteratively. In the inspiration phase, the problem (or opportunity) is explored; the ideation is the phase of the generation, development, and testing of ideas; and the implementation phase is the delivery of the project to the consumers [35].

The author describes DT as a team-based approach to innovation and defines DT as “a discipline that uses the designer’s sensibility and methods to match people’s needs with what is technologically feasible and what a viable business strategy can convert into customer value and market opportunity”, referring to some aspects to be considered for the process to succeed: involving DT from the beginning, taking a human-centred approach, using experimentation and prototypes in the early stages, using co-creation, blending big and small projects, adjusting the budget to the speed of the project, looking for an interdisciplinary team, and designing in cycles. Collaboration and integration are also essential for the design’s success so everyone can participate.

#### 3.2.4. Design Thinking as a Process

Beckman and Barry [36] intended to integrate the processes of innovation and learning in a way that could be used across disciplines. The authors relied on the model of Owen [46], who views design as a process of knowledge development that operates in the analytic and synthetic realms as well as the theoretical and practical realms and is intended to fit all fields once the proper tools are employed. The authors also explored theories of learning, citing the experimental theory of Kolb [47], where four steps of learning (experiencing, reflecting, thinking, and acting) work iteratively. The Kolb model also involves two approaches to grasping an experience (concrete experiences and abstract conceptualisation) and two others to transform the experience (reflective and active experimentation). Thus, the authors established a parallel between the theories of Kolb and Owen, creating a new model. Although the process is not linear, the authors established four stages for generating observations, frameworks, imperatives, and solutions.

Each of the steps of the model involves a different kind of reasoning and skills, as follows. (1) The observation stage involves an understanding of the context (consumers’ needs) through concrete analytical means (by observation, market research, or direct interaction with the users). (2) In the framework stage, the designers move to the abstract realm, coming up with new solutions by identifying interesting stories or behaviours and creating matrices or timelines. (3) In the imperative step, the designers synthesise the imperatives to be fulfilled. (4) In the solution step, there is a return to the concrete realm to generate the solution that best fits the imperatives and test it.

Carlgren et al. [37] approached the DT process by interviewing six companies that used it. The authors found five common characteristics: (1) a user focus, (2) framing the problem, (3) visualisation techniques, (4) experimentation, and (5) diversity. Despite these common characteristics, how each company used DT depended on their needs, sources of knowledge, and organisational context.

Waidelich et al. [38] reviewed the models used by the DT literature, finding similarities among them: the ideation phase is the most mentioned, followed by the prototype, and most models start with the understanding phase. As the authors stated, DT methods are applied to achieve the best results and, therefore, there is no standardisation; however, the similarities are related to the popularity of the IDEO model (3I) and the Stanford d.School [6,7,48].

#### 3.2.5. Design Thinking Reviews

The most relevant reviews on DT make a clear distinction between DT as part of the design theory and the concept of management [3,12,22]. Kimbell [12] attributed the change in the designers’ role to the natural evolution of time, namely the way capitalism has changed and is now seen as dynamic rather than bureaucratic; the new global economy of signs that is product-saturated; the rise of the creative class, who find meaning in work; and the request for answers after the 2008 crisis. Johansson-Skoldberg et al. [3] pointed out the differences between both discourses. For the “designers”, DT presupposes the creation of artefacts, a reflexive practice, problem-solving, a practice-based activity of making sense of things, and the creation of meaning (rather than artefacts); for the “management”, DT includes the IDEO’s way of working, a way to approach indeterminate organisational problems, and part of management theory. Johansson-Skoldberg et al. [3] concluded that both concepts are so different that they cannot be merged.

Micheli et al. [4] defined DT as an “umbrella construct” and described its characteristics through a literature review complemented by an exercise with designers. The authors identified 10 attributes of DT: (1) creativity and innovation, (2) user-centeredness and the users’ involvement; (3) problem-solving; (4) iteration and experimentation, (5) interdisciplinary collaboration, (6) the ability to visualise; (7) a gestalt view, (8) abductive reasoning; (9) tolerance of ambiguity and failure; and (10) blending rationality and intuition. The authors also found the eight most commonly used tools and methods reported in the literature: prototyping, visualisation, ethnography, experiments, brainstorming, journey maps, personas, and mind maps. From the cluster analysis, the authors found five perspectives on DT: (1) DT as interdisciplinary collaboration; (2) DT as belonging to the design field (abductive reasoning, the ability to visualise, interdisciplinary collaboration, and tolerance for ambiguity and failure); (3) DT as problem-solving and resilience (tolerance for ambiguity and failure, and interdisciplinary collaboration); (4) DT as reflecting upon the whole (the ability to visualise and a gestalt view of problems); (5) learning to think as a designer (gestalt and the role of blending analysis and intuition).

In their review, Micheli et al. [4] included the concepts of both “designers” and “managers”, meaning that both discourses can be merged, contrary to the abovementioned study by Johansson-Skoldberg et al. [3]. As an example, Lawson [10], who is a designer, considered the client or the user as generators of constraints. In the case of well-known methodologies such as that proposed by Brown [6], the presence of synthesis/analysis and convergent/divergent thinking is evident, as is the way that the author views the process as iterative, denying the step-by-step procedure, approaching his point of view from the same direction as Rowe [26].

### 3.3. Main Remarks on the Evolution of the Concept of Design Thinking 

**Figure 4:**
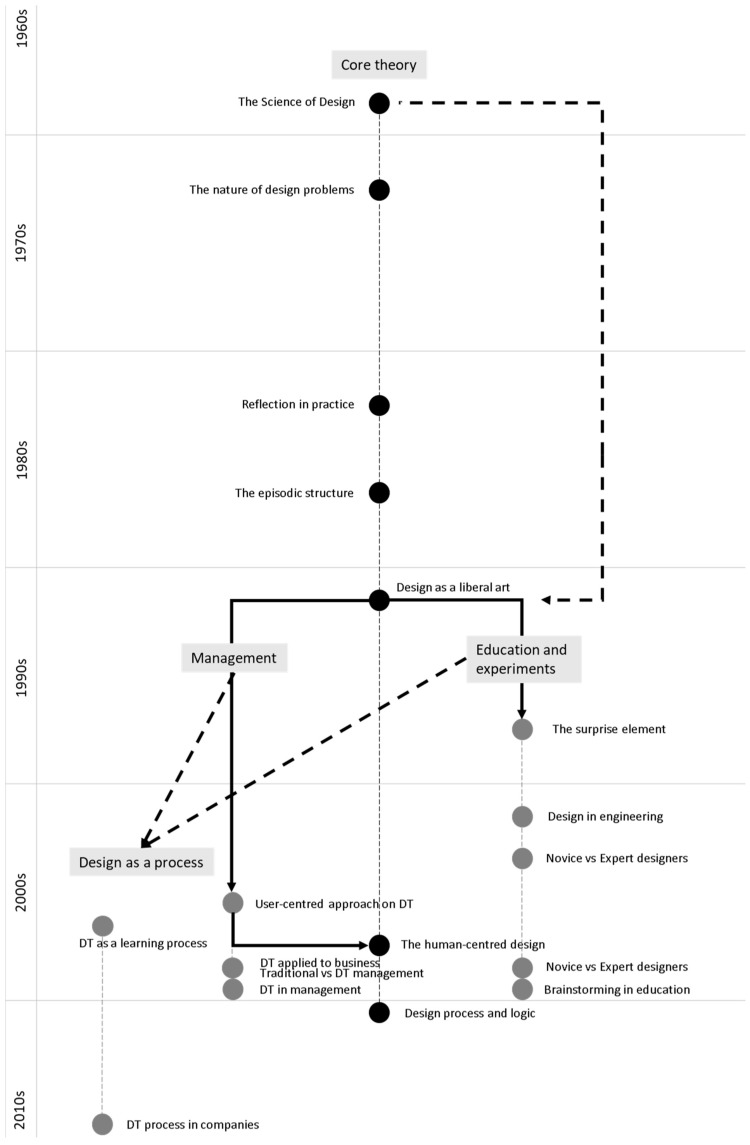
Interactions among the themes discussed by the different authors. The titles and numbers represent the reviewed works found in Table 1. The lines represent the interactions. Black dots: general Design studies; Gray dots: DT branches. Bold lines: Direct connection; Dashed lines: implicit connection. References: The science of design [11]; The nature of design problems [24]; Reflection in practice [25]; The episodic structure [26]; Design as a liberal art [8]; Human-centred design [6]; Design process and logic [27]; The surprise element [28]; Design in engineering education [29]; Novice vs. expert designers [30,31]; Brainstorming in education [32]; User-centred approach to DT [7]; DT applied to business [33]; Traditional/DT-based companies [34]; DT in management [35]; DT as a learning process [36]; The DT process in companies [37]; Biases on DT [22]; The three types of DT [12]; Defining DT [4]; The DT processes [38].

Figure 4 visually represents the relationships between the different works, helping to understand the historical progression and subdivisions within the DT field [6,8,11]. The figure reveals that the work of Buchanan [8] in 1992 served as a critical breakpoint, leading to subsequent themes related to the applications of education and management. It also demonstrates the linkage between Buchanan’s work [8] and Simons’ ideas [11], as well as the contributions from the field of management to Brown’s work [6]. The interactions among these themes are represented by lines in the figure.

This view eliminates the gap mentioned earlier. Hence, Brown’s work [6], although initially considered outside the design domain and more aligned with management, is now considered part of design theory. Despite the lack of citations in Brown’s work [6], it encompassed the core principles of design theory, also adding the perspective of management; furthermore, Brown’s work [6] consisted of an easy-reading article and presented DT as a framework that can be used easily by the industry, thus gaining more and more popularity.

Despite not explicitly mentioning the term “design thinking” in his book The Sciences of the Artificial, Simon, regarded as the father of DT, conceptualised design as the underlying knowledge behind the creation of objects [11]. The author transformed the traditional notion of the designer as a mere “doer” into that of a “thinker”, as, according to Simon, all human creations are considered “artificial” and can be designed [11]. Although some review articles proposed a division between academic design and other perspectives on DT, considering Simon’s standpoint [11] eliminates this gap. Thus, DT should integrate the discourse of design theory and other perspectives derived from the concept of evolution.

### 3.4. Part 2. Design Thinking in Food Studies

After grouping, we found two main groups: one related to the theoretical background, comprising general theory and models of FDT (*n* = 7), and the other group that covered the applications of FDT in various areas, namely sustainability (*n* = 6), health and wellbeing (*n* = 5), education (*n* = 5), and the development of food products (*n* = 6). The results are summarised in Table 2.

As some of the works were based on existing DT methods (Table 3), making a small synthesis of the ones used in this context is relevant.

#### 3.4.1. Theory and Models of Food Design Thinking 

The relationship between design and food studies becomes clear, given the context presented in Part 1. Olsen [2] was one of the first authors to discuss DT applied to food. The author emphasised the importance of consumer empathy, and consumers, traditionally viewed as validators of the product, at the end of the process, should be integrated at the beginning as a point of departure. Olsen [2] also referred to important steps of DT such as prototyping, consumer testing, and collaboration, suggesting an open innovation system where research, industry, and users could work together. In the same sense, Shimek [21] discussed the advantages of integrating DT in the process of innovation, connecting qualitative consumer studies and tangible product design; the author considered food consumers’ behaviour to be more emotional than rational and, therefore, the opportunity is not to correct the consumers but to follow their beliefs. The author highlighted the five characteristics of DT that can be useful to the development of new products in the cereal industry: (1) multidisciplinary collaboration; (2) building empathy with consumers, customers, and key stakeholders; (3) development of ideas including the consumer; (4) rapid prototyping; and (5) human-centred storytelling.

Zampollo and Peacock [49] described FDT as “creating food products, food services, or food systems, where the design methods and tools are designed specifically to facilitate reflection on the eating experience”, integrating many food-related aspects. The authors developed methods and tools dedicated to FDT, creating two new tools specifically designed for FDT: Themes for Eating Design (TED) and Thoughts for Food. TED was created on the basis of a set of food-related images and was implemented to generate and describe food experiences regarding products, rooms, meetings, atmospheres, and management control systems. Thoughts for Food appeared as a new iteration of TED and consisted of cards with an icon, a word on the front, and a description on the back. It was intended to be used the same way as TED, with the advantage of being easier to manage. The authors evaluated TED and Thoughts for Food with great results, suggesting that they opened the doors to creating the FDT discipline.

Franchini et al. [50] discussed how design processes could shape innovation and proposed the integration of the stage–gate framework [79], one of the most commonly used frameworks for the development of food products with DT, bringing together the advantages of stage–gate (e.g., increasing the speed of development) with the creativity of DT. The authors used both approaches in a company (using DT to develop ideas and stage–gate [79] to process them), concluding that the model “fails to balance exploration and exploitation”, creating internal problems regarding the management of employees and resources. Despite these results, the authors concluded that the integration of these approaches should be further researched.

Despite the existence of various DT models, other authors, such as Sijtsema et al. [51], Batat and Addis [57], and Massari et al. [53], developed their own models directed towards various food issues.

Sijtsema et al. [51] proposed an FDT model for the development of food products, departing from Olsen’s (2015) perspective. The Circular Food Design model represents the iterative process of FDT and combines the Double Diamond model [76] with elements of sustainability. This user-centred and flexible model serves as a collaborative tool. It aims to structure and integrate different perspectives and phases of innovation within the food system, promoting creativity and increasing the likelihood of success in the development of healthy and sustainable food. Massari et al. [53] also proposed a proposed a model related to sustainability. The CEASE (Communities, Engagement, Actions, Shareability, Ecosystems) model focuses on the issue of food waste and intends to maintain and consolidate the emerging strategies of food management and behaviour styles developed within the COVID-19 pandemic lockdown. The authors used the Stanford d.School [78] DT as a base, considering empathising with the communities, defining by analysing the solutions adopted by consumers to solve similar problems, ideating through co-design, prototyping by creating suitable conditions and scenarios to develop collaboration and collective action; and testing the impacts of the proposal.

Batat and Addis [52] developed a framework oriented explicitly towards food experiences and wellbeing. To create the model, the authors compared the concepts of DT, food design, and design of the food experience regarding their orientation, focus, the actors involved, the types of innovation, the outcomes, creativity level, and competitive advantages, concluding that the concepts failed to capture the experiential aspects of food consumption and neglected to integrate consumers’ wellbeing throughout the various stages of the food journey. The authors also discussed how the perspectives of producers and consumers regarding FDT differ, and the factors that influence consumers’ experiences (physical, social, and technological environments) and their overall wellbeing (life satisfaction, affect, and sense of purpose), creating an integrative model.

#### 3.4.2. Food Design Thinking and Education

Similarly to what happens in DT, FDT was also implemented in the educational context.

Mitchellet al. [54] explored the implementation of DT in Otago Polytechnic’s Bachelor of Culinary Arts (OPBCA) programme, aiming to foster “designerly ways of knowing” in graduates. The core methodology used in this programme was based on the Double Diamond approach [76]. Students engaged in project-based learning, where they received a project brief outlining the requirements and constraints of the problem to solve. Throughout the learning process, the students prototyped and tested dishes, documented their progress, and delivered the final outcomes to clients. This approach allowed students to bridge their individual experiences and interests with the professional culinary world, promoting the acquisition of fundamental cookery techniques while encouraging critical thinking and problem-solving skills.

Similarly, Frost [55] described the work performed at the Nordic Food Lab, a research and development institution that operated between 2008 and 2018. The lab was a collaborative space for chefs and researchers to explore various aspects of gastronomy, particularly molecular gastronomy. The project contributed to the field of knowledge by sharing the research findings with the community. It highlighted the potential for cross-pollination between chefs and scientists, where chefs could incorporate sensory science techniques in their restaurants, and scientists could leverage design and prototyping to expedite food innovation.

Derler et al. [56] conducted a case study using the DT approach to examine trans-disciplinarity’s role in enhancing secondary school students’ food literacy. The study included nutrition- and agriculture-focused secondary school students and a polytechnic school. The students were asked to develop sustainable food products for their peers. The research process encompassed four phases: exploration, ideation, prototyping, and optimisation. During the exploration phase, workshops were conducted, and students’ food consumption behaviours and perceptions were assessed using surveys, photovoice techniques, and food diaries. In the ideation phase, the collected data were analysed, and food product ideas were generated on the basis of personas representing typical users. The students then voted on the best ideas and created product specification sheets. In the prototyping and optimisation phase, students conducted experiments to refine their prototypes, documented their learning process, and iteratively improved their food products. The authors emphasised the DT approach’s motivational aspects and highlighted the positive influence of the researcher’s involvement from the project’s inception.

Kaygan [57] examined the development of teams within the context of a collaborative design course that integrated industrial design and food engineering students. The author underscored the significance of collaboration in addressing complex societal issues, noting the growing interest in interdisciplinary programmes. The course focused on “food away from home,” and students engaged in a DT methodology. The initial week fostered open debates on the design strategy, allowing students to identify the commonalities and differences in their fields of knowledge. Subsequently, lectures were conducted to introduce students to the theory of DT, emphasising critical thinking skills and mindset. The process was based on the Stanford d.School [78] DT model, along with key concepts such as wicked problems, user-centeredness, and the characteristics of a design thinker. Students used design tools such as mind maps and role-playing to brainstorm ideas and generate potential solutions in the following weeks. A brainstorming card tool facilitated the generation of ideas, and students sketched the selected ones. Students were encouraged to independently prototype their ideas as the project progressed, with reduced teacher assistance. The author highlighted the value of DT in introducing engineering students to a fresh perspective on design while providing design students with a simplified version of the design process.

Pärli et al. [58] presented a case study demonstrating the application of DT in creating interventions for real-world food practices within a Master’s course. The project aimed to address academic and societal needs, specifically focusing on shifting residents’ food practices towards sustainability and evaluating the effectiveness of the interventions. The students followed a three-stage process to achieve these objectives. In the initial stage, they conducted a thorough literature review, performed site observations, and conducted interviews to clarify and focus on the topic. In the subsequent stage, the students designed the interventions by combining data sources, identifying insights, and explaining the underlying reasons and methodologies behind their observations. The proposed interventions were then discussed with the community for feedback. Finally, the students implemented the interventions and assessed their effects, closing the DT cycle.

#### 3.4.3. Designing for Sustainability

The search found six works related to sustainability. Three of them developed technological improvements to increase people’s awareness of the food they buy or store [59,60,61].

Nguyen et al. [59] used the DT approach to design an interactive prototype of a fridge to promote the prevention of food waste. The authors used the HPI model [77], supported by various tools and techniques. In the understanding and ideation phases, the authors conducted surveys and semi-structured interviews to investigate users’ behaviours and habits regarding food storage. The research revealed three main concerns: consumers’ lack of awareness of the food in their fridge, a lack of awareness of food quality, and a lack of interest in eating leftovers. To address these concerns, the authors prototyped objects such as stickers (for categorising food types), sliders with timers (for categorising the states of food), and an LCD display that reacted to the state of the food, making the fridge more engaging and encouraging cooking. The prototype was evaluated with 10 participants through semi-structured interviews, indicating its effectiveness in helping users categorise food, making the fridge more interesting to use, raising awareness of the quality of food and motivating users to reduce food waste. Bonaccorsi et al. [65] developed an innovative smart freezer using an industrial DT approach focused on the users’ involvement. The authors collaborated with 11 users to recreate typical conditions of domestic freezer use and evaluated existing models of fridges. Contextual inquiry, observation, and thinking aloud were used in a heuristic approach. The outcomes of the assessment phases and the user involvement stage provided insights into user–product interactions and the users’ needs, which informed the prototype’s design proposal and technological developments.

Kamil et al. [61] used a DT approach to develop a system (reader and app) to address the problem of food waste. Through interviews with 52 users, the authors identified five elements of the solutions. The idea generation phase involved brainstorming sessions to establish the design criteria, including simplicity, user-friendliness, interactivity, emotional appeal through aesthetics, and the use of quality materials. Various solutions were depicted through sketches of the object–user interface, and a 3D design was created to visualise the selected solution. A prototype was developed using a 3D printer. The Pebble system was implemented in supermarkets, allowing users to evaluate it to acquire food-related data and expiration dates, raising their awareness of the food they bought and its quality.

On a larger scale, Purnomo et al. [62] and Adebayo et al. [63] collaborated with farmers, helping them to deal with imbalances in food production regarding excess or a lack of production. Purnomo et al. [62] conducted participatory action research using a DT approach to address the issue of excess taro production in an Indonesian village. The researchers followed their own six-stage process: meeting with community leaders, visiting gardens, empathising with the community, and identifying the challenges related to the processing and commercialisation of taro. Based on their findings, the researchers developed a business model focusing on taro flour as a derivative product. The ideal business model involved key partners such as farmers, local non-governmental organisations, and local governments. This model aimed to establish a structured and clear pathway for the business to thrive.

Adebayo et al. [63] studied the needs of African farmers and used a DT method to improve farms’ productivity. The authors implemented the Stanford d.School model [78], supported by semi-structured interviews and observations, brainstorming sessions with farmers, sketches, scenarios, and mini-prototypes. The proposed solution involved placing sensors on farm fields connected to social media platforms to monitor environmental changes such as temperature, relative humidity, soil moisture, pH, and pest levels. The solution was evaluated and demonstrated its effectiveness in helping farmers improve crop yields.

In another approach to food-related sustainability issues, Asawadechsakdi et al. [64] used a DT-based approach to create single-use packaging from leaves to reduce plastic bag waste. The authors followed their own four-phase model (identification of the problem, understanding consumers’ needs, creativity, and prototyping), supported by interviews with vendors and consumers, observations of package sizes, cooking methods, carrying behaviours, container-opening behaviours, and eating methods. The process of creativity was based on brainstorming sessions and morphological analysis to separate the attributes and functions of the packaging, inspiring different concepts. The authors focused on moulding leaves and stabilising the package, experimenting with various techniques. Two design types were developed: a fresh leaf package with a hard frame and a dried leaf package with a soft frame. After evaluating the designs through interviews with voluntary package users, the fresh leaf package was selected due to its ability to support the food’s weight, carry dry and moist food, and be held with one hand.

These studies illustrate the application of DT in addressing food waste and environmental concerns through the design of innovative prototypes and systems. By involving users in the design process and considering their needs and behaviours, these approaches contribute to reducing food waste, promoting sustainable practices and improving the overall efficiency of food-related systems.

#### 3.4.4. Design Thinking for Health and Wellbeing

This section includes five studies. Mummah et al. [65] and Bogomolova et al. [66] used the DT approach to motivate healthy eating; Fernhaber et al. [67] and Kalita et al. [68] worked on external problems that could compromise healthy eating, namely the case of access to food and the case of food hygiene. Finally, Rejikumar et al. [69] worked on improving food-related wellbeing during restaurant service.

Mummah et al. [65] developed an app to motivate healthy eating among overweight adults using the DT approach. The IDEAS framework guided the iterative process, consisting of eight phases. In the “empathise” phase, the authors conducted semi-structured interviews to understand the target users’ struggles with meal planning and their goals of building a healthier lifestyle. The “target behaviour” phase involved reviewing the literature to identify eating behaviours with health benefits, such as increasing consumption of fruits and vegetables. Grounded in behavioural theory, the authors reviewed the relevant literature to inform the design process. In the “ideate” phase, brainstorming sessions were held in a multidisciplinary environment to generate potential features of the mobile app. Prototypes of potential products were sketched and discussed, resulting in two prototypes. Users’ feedback on the prototypes was gathered through interviews, which helped refine the design. The minimum viable product was then built, focusing on the users’ experience, the visual design, logic, and content. The potential efficacy and usability of the app were evaluated through a questionnaire, measuring usability and users’ satisfaction. The resulting app, Vegethon, is a standalone mobile app that allows users to self-monitor their consumption of vegetables, incorporating elements of gamification to reward the process of behavioural change. The users can set personalised goals, log their consumption of vegetables, and receive challenges, points, and notifications to reinforce their goals. The app also provides advice, tips, and a personalised weekly report.

Bogomolova et al. [66] developed a programme called “A Healthy Choice” to encourage healthier food purchases in an Australian supermarket. The research consisted of two parts. In the first part, co-design sessions were conducted with consumers and supermarket staff. These sessions followed a co-design process, including word association tasks, group discussions, reviews of former programmes, generation of ideas, and pitching ideas. The participants collaboratively created proposals and pitched their ideas. In the second part, the proposals from the co-design sessions were evaluated by the supermarket management for feasibility. The “A Healthy Choice” programme was developed, implemented, and evaluated. The programme involved strategies suggested by consumers and addressed the product, physical evidence, and dimensions of the place of the marketing mix. Shelf talkers with the “A Healthy Choice” logo and descriptions were placed on selected healthy products in the store. The program was evaluated on the basis of consumption data collected through surveys and sales of healthy items, showing positive results.

Fernhaber et al. [68] used a participatory design process based on the “community INNOVATE” DT framework to address the issue of food deserts in the USA. The process involved engaging various stakeholders to co-create solutions for improving access to healthy and affordable food. The process was divided into four phases. In the inquiry phase, the authors interacted with the community through conversations, workshops, and an online platform to understand the root problems and prioritise the major areas of concern. The ideation phase focused on generating ideas for addressing the identified goals through brainstorming sessions with the community, then narrowing down the ideas on the basis of their quality. In the investigation phase, community members developed solutions to shape the ideas. Finally, actors could pitch their initiatives in the impact phase and network for grants to implement the selected ideas. The community INNOVATE process facilitated collaborative problem-solving and empowered community members to play an active role in finding solutions to the challenges of access to food.

Kalita et al. [68] applied the DT approach to improving the economics and hygiene of street food vending in India. A team of management and design students used visual ethnographic narratives to study users’ behaviour and issues of vending through marketing research. Sketches and simple prototypes were used to generate and address specific design problems. The product detailing was modelled in 3D, and a 1:1 scale prototype was developed. Various materials and forms of edible packaging and serving were explored. The prototypes underwent usability and feasibility testing, including focus group discussions.

Rejikumar et al. [69] proposed a methodology to create a service design that enhances the dining experience in a restaurant in India. The authors combined the Stanford d.School DT model [78] with the Taguchi method to identify optimal settings for selected design attributes contributing to food-related wellbeing. Customers’ objections and concerns in restaurant dining experiences were collected in the “empathise” stage. The customers’ needs were defined, and brainstorming sessions were conducted with experts to generate ideas and create multiple options for the selected attributes of the service design. Scenario-based narrations of the prototype designs were used to facilitate customers’ evaluations. The robustness of the prototype designs was evaluated through a Taguchi experiment. The research revealed that perceptions of hygiene and service design are critical in food-related wellbeing. Consumers valued information about the ingredients and the recipes’ transparency, which enhanced their food-related wellbeing.

These studies demonstrated the application of the DT approach in promoting health and wellbeing, addressing issues such as motivation towards healthy eating, access to healthy food, and the improvement of dining experiences. Through the users’ involvement, collaborative problem-solving, and iterative design processes, these approaches can enhance individuals’ health behaviours and overall wellbeing.

#### 3.4.5. Design Thinking for the Development of Food Products 

Bunyamin et al. [70] used the DT approach to develop food products in collaboration with the community. The study focused on the Pikul Pengajid Indigenous Forest, known for its high productivity of tengkawang fruit oil. However, the consumption of green butter derived from the oil was low due to a lack of awareness and dislike for its pungent smell. The researchers conducted participatory action research with the community, using various tools and instruments for the collection, processing, and analysis of the data. They followed the stages of DT, including direct observation of the green butter-making process to identify pain points and gains. Based on these observations and using the constraints of criteria, they designed food products such as soft ginger cookies, tengkawang sticks, tengkawang doughnuts, tengkawang noodles, and tengkawang pie. The products were named in collaboration with naming experts to promote the region and highlight the quality and taste. An action plan was created, considering the capacity for production, diversification of derivative products, development of business groups, and the establishment of centres for processed tengkawang products.

Hastie et al. [71] used the DT approach to promote the consumption of aged cull ewe meat in Australia, which tends to become tougher as animals age. The methodology consisted of four stages. In the stage of defining the problem, stakeholders, including consumers, producers, and food service representatives, were consulted to understand their needs. In the stage of dish ideation and prototype development, 15 food service professionals received the collected information, creating 17 stimuli as images or prototypes. In phase three, two panels of participants discussed the stimuli, leading to a perceptual mapping with axes representing economy–premium and familiar–traditional concepts. The final stage involved serving eight selected dishes to a consumer panel, who rated the dishes in terms of liking, premiumness, frequency of consumption, and suitability for food service outlets. The results indicated that consumers liked the meals and considered them premium, demonstrating the success of the DT approach in addressing the problem.

Tkaczewska et al. [72] proposed a protocol for designing food products with functional properties using the DT approach, focusing on developing an antioxidant-rich snack. The protocol consisted of multiple steps, including identifying the target consumer group, diagnosing the problem, identifying its cause, creating a concept of the food product, proposing food products, preparing preliminary prototypes, creating a product description sheet, developing a detailed recipe and production process, testing and modifying the prototype, determining the nutritional value and storage conditions, and finalising the product’s characteristics and the necessary documentation (GMP/GHP and HACCP guidelines). The authors acknowledged that the protocol should be adapted, as the DT process is not linear.

Gallen et al. [73] applied the DT method to improve the experiences associated with insect-based foods. Their multidisciplinary team, including design students and specialists in various areas of knowledge, followed a three-step approach: exploration, ideation, and experimentation. In the exploration phase, data on consumption trends, the acceptance of different insect species, and acceptance of shapes were collected from the existing literature on consumers’ behaviour. In the ideation phase, the team generated 12 prototypes through brainstorming, with each group representing a persona based on emerging trends. Visual representations of the concepts of the product were created. The concepts were evaluated through recipe prototypes in the experimentation phase, and packaging designs were developed accordingly. The team presented the products to 30 consumers in guided interviews, allowing for refinement of the product based on consumers’ feedback.

Castanho et al. [75] adapted a well-known questionnaire from the area of food consumption studies to the Portuguese culture, specifically focusing on the consumption of rice, to understand the motives behind the Portuguese choice of rice, as suggested by a study in the field [80]. The authors applied the 3I/IDEO DT method. In the inspiration phase, they gathered information from the literature and consumers to design an image-based tool; in the ideation phase, consumers used this tool to generate expressions about their rice preferences; in the implementation phase, the authors developed the adapted questionnaire by comparing it with the original version. The resulting questionnaire was applied to consumers and analysed statistically. The authors emphasized the importance of using images to indirectly trigger individual responses and involving consumers throughout the process to minimize researcher bias.

Castanho et al. [74] designed a creative tool for generating ideas during the ideation phase of the development of food products, comparing its effectiveness with simple brainstorming. To design the tool, the authors collaborated with professional chefs, who provided meal recipes, concepts, and ingredients for rice-based dishes. These inputs were presented to groups of students in a card game format, where the students were asked to pick random cards from different categories and to use them as a starting point for brainstorming. At the end of the exercise, the chefs evaluated the generated ideas for adequacy, clarity, workability, and novelty. The results indicated that the ideas produced using the tool scored higher in novelty compared with those generated through simple brainstorming.

These examples highlight the application of the DT approach in the practical development of food products, involving collaboration with the community, addressing consumers’ preferences, and incorporating iterative design processes. By using DT methodologies, researchers could generate innovative food products, overcome barriers to consumption, and create solutions catering to the consumers’ needs and preferences.

### 3.5. Main Remarks on the Concept of Food Design Thinking 

DT has emerged as a valuable approach in food studies, offering innovative and user-centred solutions to various challenges. In this sense, all the practical applications of DT start with a problem, suggesting the creation of artefacts to solve it. This common objective of FDT is consistent with the theory of DT, which dates back to Simon’s description of design as the creation of artefacts to solve problems [11]. Additionally, Rittel and Webber [24] proposed the concept of design problems, further aligning with the objectives of FDT.

The application of this approach in different food-related contexts, including product development, sustainability, education, and health and wellbeing, which suggests that DT can be applied to a diverse range of food-related problems. This parallels Simon’s ideas [11] and Buchanan’s concept of DT as a problem-solving approach that can be used across various domains and subdomains [8].

Another common aspect found in the various theories and applications of FDT is to use a step-by-step process and, in some cases, mentioning iterations. These references can be attributed to the works of Schön [25] and Rowe [26], as they defined the design process as being driven by episodes and iterative steps. The step-by-step logic was also evident in work related to DT methods, which primarily divided the process into three main phases: inspiration, ideation, and implementation (3I) [6]. While these phases can be expanded indefinitely, they form the foundation of the FDT process.

Furthermore, the consumers’ involvement through empathy, understanding, and co-creation is emphasised, which is related to the most recent literature on DT, which considers the entire human-centred process. This human-centeredness is evident in the application of all areas of food design, whether the user is a consumer, a client, or society. The involvement of the end-users and stakeholders is crucial to creating solutions that address their needs effectively. Prototyping and testing were also commonly used in the studied works, as they allow for the exploration and refinement of ideas.

Despite these overall commonalities, the differences between FDT approaches can be defined by the specific problems they address, which may require alternative methodologies to the typical well-defined step-by-step process. The socioeconomic context and cultural influences are also crucial factors when implementing FDT. These factors contribute to the uniqueness of the DT process, even though there are commonalities among the different approaches.

The commonalities across different DT methods can be observed in Table 2, mainly reflected in their step-by-step approach, as discussed above. However, Table 3 shows that FDT authors often use their own models rather than a common approach, demonstrating the need for adaptation to specific realities.

## 4. Conclusions

The design thinking approach, with its evolving nature and diverse perspectives, has proven to be a versatile methodology that is applicable across various domains. While this approach is well-established in fields such as management and education, the food sector remains somewhat hesitant to adopt it, as evidenced by the limited number of articles published in scientific databases.

This review study demonstrates that design thinking can be effectively applied within the food industry, offering substantial benefits. Furthermore, as the core principles of design thinking—user-centred design, collaboration, and prototyping—are applicable across different contexts, this approach can be used in various subfields, such as health and wellbeing or the development of food products, providing new perspectives and opportunities for innovation in this area.

Even though there are different views on how design thinking should be applied, its development shows the importance of combining different perspectives, understanding complex issues, and working across disciplines. As the field continues to grow, design thinking is likely to remain an important tool for solving complex problems. Further research and practical work will probably reveal new ways to use design thinking, advancing its role in the food industry.

## Figures and Tables

**Table 1:** Reviewed documents grouped by theme, including publication date, citation count, and main subject discussed.

Author [Reference]	Publication Date	Citation Count	Main Contribution
Design Thinking			
Simon [11]	1969	195	The science of design
Rittel and Webber [24]	1973	192	The nature of design problems
Schön [25]	1983	199	Reflection in practice
Rowe [26]	1987	164	The episodic structure
Buchanan [8]	1992	312	Design as a liberal art
Brown [6]	2008	582	Human-centred design
Dorst [27]	2011	280	Design process and logic
Education and experiments			
Dorst and Cross [28]	2001	146	The surprise element
Dym et al. [29]	2005	228	Design in engineering education
Cross [30]	2007	152	Novice vs. expert designers
Razzouk and Shute [31]	2012	179	Novice vs. expert designers
Seidel and Fixson [32]	2013	112	Brainstorming in education
Design thinking and management			
Dunne and Martin [7]	2006	190	User-centred approach to DT
Brown [33]	2009	307	DT applied to business
Martin [34]	2009	228	Traditional/DT-based companies
Brown and Wyatt [35]	2010	173	DT in management
Design as a process			
Beckman and Barry [36]	2007	144	DT as a learning process
Carlgren et al. [37]	2016	117	The DT process in companies
Reviews			
Liedtka [22]	2015		Biases on DT
Kimbell [12]	2011		The three types of DT
Micheli et al. [4]	2019		Defining DT
Waidelich et al. [38]	2018		The DT processes

**Table 2:** Reviewed works on Food Design Thinking grouped by theme (in bold), their theoretical implications, the problem or product worked on, and the method used.

	Main Contributions/Objectives	DT Method
	**Theory and models**	
Olsen [2]	Integrating the consumers from the beginning	N.a.
Zampollo and Peacock [49]	Describing the concept of FDT and generating new tools	N.a.
Franchini et al. [50]	Using stage-gate and FDT simultaneously	N.a.
Shimek [21]	Characterising FDT for product development	N.a.
Sijtsema et al. [51]	Designing a non-linear FDT model directed to sustainability	Double diamond
Batat and Addis [52]	Designing an FDT model to improve the food experience	Own model
Massari et al. [53]	Designing an FDT model directed at preventing food waste	Stanford d.School
	**Food Design Thinking and education**	
Mitchell et al. [54]	Implementing FDT	Double diamond
Frost [55]	Integrating science and culinary arts	N.a
Derler et al. [56]	Comparing FDT with traditional food development	3I/IDEO
Kaygan [57]	Integrating industrial design with food engineering	Stanford d.School
Pärli et al. [58]	Shifting food practices towards sustainability	Own model
	**Designing for sustainability**	
Nguyen et al. [59]	Developing an interactive fridge to prevent food waste	HPI
Bonaccorsi et al. [60]	Developing a smart freezer	3I/IDEO
Kamil et al. [61]	Developing a technological system to prevent food waste	Own model
Purnomo et al. [62]	Solving the problem of excess taro production	Own model
Adebayo et al. [63]	Improving farm productivity	Stanford d.School
Asawadechsakdi and Chavalkul [64]	Developing a sustainable single-use package	Own model
	**Design thinking for health and wellbeing**	
Mummah et al. [65]	Developing an app to motivate healthy eating	Own model
Bogomolova et al. [66]	Developing a plan to encourage healthier food purchases	Own model
Fernhaber et al. [67]	Developing a plan to deal with food deserts	Own model
Kalita et al. [68]	Developing a plan to improve street food vending hygiene	Own model
Rejikumar et al. [69]	Enhancing the dining experience	Stanford d.School
	**Design thinking for development of food products**	
Bunyamin et al. [70]	Developing foods with Tengkawang oil	Own model
Hastie et al. [71]	Developing foods to promote consumption of aged meat	Own model
Tkaczewskaet al. [72]	Developing food products with functional properties	Own model
Gallen et al. [73]	Developing insect-based foods and their packaging to improve the consumers’ experience	3I/IDEO
Castanho et al. [74]	Creating a structured technique for idea generation for the development of innovative rice-based products	Own model
Castanho et al. [75]	Adapting a questionnaire to the cultural background and a particular food to inform product development	3I/IDEO

**Table 3:** Design Thinking models used in the reviewed works.

Popular Designation	Phases
Double diamond [76]	Discover Define Develop Deliver
HPI model [77]	Understanding Observing Point of view Finding ideas Prototyping Testing
3I (IDEO) [6]	Inspiration Ideation Implementation
Stanford d.School [78]	Empathise Define Ideate Prototype Test

## Data Availability

No new data were created or analysed in this study. Data sharing is not applicable to this article.
